# Hydrogel-based strategies for periprosthetic joint infection: from osteoimmune mechanisms to clinical translation

**DOI:** 10.3389/fcell.2026.1911893

**Published:** 2026-08-17

**Authors:** Chao Zhang, Rui Zhang, Yuwen Yang, Ye Wang, Junxue Wu

**Affiliations:** 1 Department of Orthopedics, Affiliated Hospital of North Sichuan Medical College, Nanchong, Sichuan, China; 2 Department of Integrated Traditional Chinese and Western Medicine of Colorectal and Anal Diseases, Affiliated Hospital of North Sichuan Medical College, Nanchong, Sichuan, China; 3 Trauma Medical Center, Department of Orthopaedic Surgery, West China Hospital, Sichuan University, Chengdu, China; 4 Department of Orthopaedics, West China Hospital, Sichuan University, Chengdu, China

**Keywords:** biofilm, clinical translation, controlled release, hydrogels, implant interface, osteoimmunity, periprosthetic joint infection

## Abstract

Periprosthetic joint infection (PJI) is sustained by implant-associated biofilms, ineffective immune activation, oxidative stress, and impaired osseointegration, and therefore requires more than local bacterial killing alone. Hydrogel-based therapies have attracted growing attention, but their biological effects are often attributed indiscriminately to the hydrogel even when they are primarily produced by incorporated antibiotics, antimicrobial peptides, enzymes, nanoparticles, growth factors, or cells. This narrative review develops a material-centered framework that distinguishes three sources of therapeutic activity: matrix-intrinsic functions, payload-derived biological effects, and hydrogel-enabled effects. We summarize the interface-specific pathophysiology of PJI and analyze the hydrogel properties most relevant to peri-implant treatment, including local retention, injectability, conformability to irregular dead space, wet-surface and implant-interface adaptation, tunable degradation, controlled or stimuli-responsive release, and extracellular matrix-like support. Representative antibacterial, antibiofilm, redox-regulating, immunomodulatory, angiogenic, and osteogenic systems are assessed according to their material composition, payload, release mechanism, experimental model, and direct relevance to true PJI. Hydrogels are also compared with polymethylmethacrylate cement, calcium sulfate carriers, nanoparticles or microspheres, electrospun scaffolds, and conventional implant coatings to clarify their scenario-specific advantages and limitations. The available evidence indicates that hydrogels are most valuable when they improve spatial retention, temporal sequencing, interface contact, or compatibility with labile therapeutics; they are not universally superior when mechanical support, established surgical familiarity, or long-term structural stability is required. Translation will depend on clinically representative implant-associated models, standardized reporting, sterilization and storage compatibility, reproducible manufacturing, surgical usability, and proportionate regulatory complexity. This hydrogel-enabled perspective supports indication-driven design and more rigorous evaluation of local biomaterial strategies for PJI.

## 1 Introduction

PJI remains one of the most challenging complications following total joint arthroplasty. Although its overall incidence is relatively low, PJI is associated with prolonged antimicrobial treatment, repeated surgical intervention, substantial bone and soft-tissue loss, impaired joint function, and considerable socioeconomic burden (Patel, 2023; Aftab et al., 2025). Depending on infection chronicity, implant stability, pathogen characteristics, and host condition, current treatment may involve debridement, antibiotics, and implant retention (DAIR), one-stage revision, two-stage revision, or salvage procedures (Karachalios and Komnos, 2021). These approaches remain the foundation of clinical management, but treatment failure continues to occur because PJI is sustained by more than the presence of planktonic microorganisms.

Bacterial adhesion to the prosthetic surface initiates extracellular polymeric substance (EPS) production and biofilm maturation. Biofilm architecture restricts antimicrobial penetration, reduces bacterial metabolic activity, supports persister populations, and limits effective immune clearance (Flemming et al., 2016; Sauer et al., 2022; Akay and Yaghmur, 2024). Neutrophils and macrophages can remain continuously activated without eradicating biofilm-embedded bacteria, leading to persistent release of reactive oxygen species (ROS), proteases, cytokines, and neutrophil extracellular traps (NETs). The result is inflammation without resolution and progressive damage to the peri-implant tissues (Visperas et al., 2022; Campoccia et al., 2023).

The consequences extend beyond infection. Sustained inflammatory and oxidative signaling impairs mesenchymal stromal cell viability, suppresses osteoblast differentiation, promotes osteoclastogenesis, compromises vascular support, and weakens osseointegration (Bandick et al., 2023; Kushioka et al., 2023; Piuzzi et al., 2024). PJI can therefore be viewed as a biofilm-driven disorder of the implant-tissue interface in which microbial persistence, osteoimmune dysregulation, mechanical instability, and regenerative failure reinforce one another.

This interface-centered pathology imposes demanding design requirements on local treatment. A clinically useful material should remain at the target site, contact irregular peri-implant spaces, tolerate blood and wound fluid, deliver an appropriate local dose, limit systemic exposure, and preserve compatibility with cells responsible for bone repair. Depending on the indication, it may also need to prevent early adhesion, penetrate or disrupt mature biofilms, respond to acidity or elevated ROS, and later support inflammatory resolution and tissue reconstruction (Dini et al., 2025).

Hydrogels are highly hydrated three-dimensional polymer networks whose crosslinking, porosity, degradation, adhesiveness, and mechanical properties can be tuned for specific applications (Li and Mooney, 2016; Ho et al., 2022; Cao et al., 2024). Their potential in PJI arises primarily from local retention, injectability, conformability, wet-surface adaptation, implant-interface coating, and control of payload release. Mild fabrication conditions also permit encapsulation of heat-sensitive antibiotics, antimicrobial peptides (AMPs), enzymes, proteins, cells, extracellular vesicles, and nanoparticles. The defensive antibacterial coating (DAC), a resorbable hydrogel used for intraoperative antibiotic delivery at the implant interface, provides an important clinical example (Romanò et al., 2015; Pressato et al., 2023).

The origin of biological activity must nevertheless be interpreted carefully. Antibiotics, AMPs, DNase, metal ions, nanozymes, growth factors, cells, and extracellular vesicles frequently provide the dominant antibacterial or regenerative function, whereas the hydrogel controls localization, protection, diffusion, and release. Some matrices do possess intrinsic antibacterial, anti-fouling, antioxidant, adhesive, or immunomodulatory properties, but these effects should be separated from payload-derived activity. A third category comprises therapeutic synergy enabled specifically by hydrogel architecture, including prolonged retention, co-localization, sequential delivery, infection-triggered release, and spatial confinement of potentially toxic treatments (Cao et al., 2021; Kalelkar et al., 2021; Choi et al., 2023).

Accordingly, this review evaluates hydrogel-based PJI therapies through three complementary levels: matrix-intrinsic functions, payload-derived effects, and hydrogel-enabled effects. We first outline the interface pathophysiology of PJI, then examine hydrogel-specific design principles, representative anti-infective and osteoimmune strategies, evidence relevance to true PJI, comparison with alternative local platforms, and barriers to clinical translation. The objective is to identify where hydrogels provide a genuine material advantage, where their superiority remains uncertain, and how future systems can be aligned with defined surgical indications rather than maximal multifunctionality.

### 1.1 Literature search strategy

This article is a narrative review rather than a systematic review. PubMed, Web of Science, and Embase were searched from database inception to 31 May 2026 using combinations of the terms “periprosthetic joint infection,” “prosthetic joint infection,” “implant-associated infection,” “hydrogel,” “biofilm,” “antibiofilm,” “osteoimmunity,” “macrophage polarization,” “neutrophil extracellular traps,” “reactive oxygen species,” “bone regeneration,” “angiogenesis,” “implant coating,” and “local drug delivery.” Reference lists of relevant reviews and representative original studies were also screened.

Priority was given to peer-reviewed studies that reported hydrogel composition, release or activation mechanisms, implant-associated infection models, mechanistic osteoimmune outcomes, or clinical translation. Clinical PJI studies and *in vivo* implant-associated infection models were considered the most direct evidence. Osteomyelitis, infected bone-defect, wound-infection, and non-infected bone regeneration models were included when they clarified material design or biological mechanisms, but they were not interpreted as equivalent to PJI evidence. Because the literature was selected narratively without a preregistered protocol or quantitative synthesis, the review remains susceptible to selection bias and heterogeneity in materials, pathogens, experimental models, and outcome measures.

## 2 Pathophysiological basis of PJI

PJI is a complex pathological condition characterized by the dynamic interplay among microbial persistence, host immune responses, and impaired tissue regeneration (Zimmerli et al., 2004). Unlike acute infections that can often be resolved through effective antimicrobial therapy, PJI frequently progresses into a chronic and recalcitrant state. This progression is driven by the establishment of biofilms on implant surfaces, the persistence of an ineffective yet sustained inflammatory response, and disruption of bone remodeling processes required for stable osseointegration. Importantly, these processes are not independent; rather, they constitute a self-reinforcing pathological loop that perpetuates infection and hinders tissue repair.

### 2.1 Initial bacterial adhesion and biofilm formation on implant surfaces

The pathogenesis of PJI is initiated by bacterial adhesion to the implant surface, a process governed by both material characteristics and host-derived conditioning films. Within minutes after implantation, host proteins such as fibrinogen, fibronectin, and albumin adsorb onto the implant surface, forming a bioactive layer that facilitates bacterial attachment (Rima et al., 2024). Pathogens, particularly *Staphylococcus aureus* and *Staphylococcus* epidermidis, express surface adhesins of the MSCRAMM family (microbial surface components recognizing adhesive matrix molecules) that specifically recognize these host proteins, thereby enabling stable colonization of orthopedic biomaterials (Maurin et al., 2021; Camaione et al., 2025).

Following initial adhesion, bacteria proliferate and secrete EPS, leading to the formation of a structured biofilm. This matrix not only provides mechanical stability but also establishes chemical gradients that protect bacteria from environmental stressors (Flemming et al., 2016; Lu et al., 2022). Within biofilms, bacterial populations exhibit pronounced phenotypic heterogeneity, including metabolically inactive persister cells that display high tolerance to antibiotics; these persisters are believed to be a principal driver of the multidrug tolerance characteristic of biofilm infections (Ciofu et al., 2022). As a result, biofilm-associated bacteria can survive antimicrobial treatments that are otherwise effective against planktonic cells.

The implant surface creates a permissive microenvironment for biofilm development. Unlike native tissues, implants are avascular and lack effective immune surveillance, allowing adherent bacteria to evade host clearance and establish persistent infection. Classic experimental studies showed that the mere presence of an implant prevented peritoneal host defenses from eliminating bacteria, even in previously immunized animals, underscoring how the implant interface fundamentally alters host–pathogen interactions (Arciola et al., 2018). Biofilm formation on these surfaces shelters bacteria and actively encourages persistence of infection by eluding innate and adaptive host defenses as well as antibiotic chemotherapy (Wagner and Hänsch, 2015). Consequently, early colonization events are critical determinants of disease progression, highlighting the importance of strategies aimed at preventing initial adhesion or disrupting early biofilm formation.

### 2.2 Biofilm-driven immune escape and chronic inflammation

Once established, biofilms fundamentally reshape host–pathogen interactions at the implant–tissue interface. Rather than triggering an effective and self-limiting immune response, biofilm-associated bacteria induce a persistent but functionally inefficient inflammatory state (Sauer et al., 2022; Visperas et al., 2022). This phenomenon is closely related to the structural and biological properties of biofilms. The extracellular polymeric substance matrix acts not only as a physical barrier that restricts antimicrobial penetration, but also as an immunological shield that limits direct contact between immune cells and embedded bacteria (Ciofu et al., 2022). As a result, neutrophils and macrophages are continuously recruited to the peri-implant region, yet their capacity to penetrate the biofilm architecture, phagocytose bacteria, and achieve effective microbial killing remains severely compromised.

This ineffective immune engagement gives rise to a characteristic paradox in PJI: inflammatory activity remains high, whereas bacterial clearance remains incomplete. Neutrophils may undergo prolonged activation and release reactive oxygen species, proteases, and extracellular traps, but these responses are often insufficient to eradicate biofilm-protected bacteria and may instead aggravate local tissue damage (Pettygrove et al., 2021). Similarly, macrophages exposed to persistent bacterial antigens and biofilm-derived signals tend to maintain a pro-inflammatory phenotype rather than transition toward a reparative state. This creates a form of “frustrated” innate immunity, in which immune cells remain activated but fail to resolve infection. Biofilms further reinforce this state by impairing phagocytosis, altering antigen presentation, modulating cytokine signaling, and releasing bacterial products that continuously stimulate pattern-recognition receptors. Therefore, biofilms should not be regarded merely as passive bacterial aggregates; they function as active regulators of the local immune microenvironment.

Chronic inflammation in PJI is therefore not simply a downstream consequence of infection, but a central pathological driver that links microbial persistence to tissue destruction. Sustained production of pro-inflammatory cytokines, including tumor necrosis factor-α (TNF-α) and interleukin-1β (IL-1β), perpetuates immune cell recruitment, amplifies oxidative stress, and disrupts the normal transition from inflammation to repair (Visperas et al., 2022). Under physiological conditions, early inflammation is necessary for host defense and tissue remodeling; however, in PJI, this phase fails to resolve. The persistence of inflammatory signaling suppresses mesenchymal stromal cell viability and osteogenic differentiation, impairs osteoblast function, and promotes osteoclast activation (Kushioka et al., 2023). Consequently, the peri-implant environment shifts from a regenerative niche toward a catabolic and tissue-destructive state.

This dysregulated immune environment has important implications for PJI treatment. Even when antimicrobial therapy reduces part of the bacterial burden, unresolved inflammation and biofilm-derived immune stimulation may continue to compromise bone regeneration and osseointegration. Thus, therapeutic failure in PJI cannot be explained solely by inadequate bacterial killing. It reflects a broader breakdown of the osteoimmune microenvironment, in which biofilm persistence, ineffective immune activation, chronic cytokine production, and impaired bone repair form a self-reinforcing pathological loop (Kushioka et al., 2023). From this perspective, effective intervention should not only target biofilm eradication, but also restore immune resolution and create conditions favorable for peri-implant bone regeneration.

### 2.3 Infection-associated bone destruction and impaired osseointegration

Concomitant with microbial persistence and chronic inflammation, the peri-implant bone microenvironment undergoes profound pathological alterations (Qin et al., 2024). Under physiological conditions, bone remodeling is maintained through a tightly coordinated balance between osteoblast-mediated bone formation and osteoclast-mediated bone resorption. This balance is essential for preserving bone mass, repairing microdamage, and maintaining stable osseointegration at the implant–bone interface. In PJI, however, this regulatory equilibrium is disrupted by persistent bacterial stimulation, sustained inflammatory signaling, and oxidative stress. As a result, bone remodeling shifts from a balanced regenerative process toward a catabolic state characterized by impaired bone formation and excessive bone resorption (Hinz et al., 2023). Inflammatory mediators and reactive oxygen species directly compromise the osteogenic compartment. Mesenchymal stromal cells (MSCs), which serve as key progenitors for bone repair, exhibit reduced viability, impaired recruitment, and diminished osteogenic differentiation under inflammatory conditions (Liu et al., 2018). Persistent exposure to cytokines such as TNF-α and IL-1β can suppress osteogenic lineage commitment and reduce the expression of osteogenesis-related markers, thereby limiting the formation of new bone matrix (Lacey et al., 2009). Osteoblasts are similarly affected: chronic inflammation impairs their differentiation, matrix-producing capacity, and mineralization function. Consequently, even when a structural defect is present and repair is biologically required, the local microenvironment is unable to support effective bone formation. At the same time, inflammatory and oxidative cues promote osteoclast activation and enhance bone resorption. Pro-inflammatory cytokines increase osteoclast differentiation and activity, partly through pathways associated with receptor activator of nuclear factor-κB ligand (RANKL) mediated osteoclastogenesis (Zhou et al., 2022). Excessive ROS further amplifies this process by disturbing redox homeostasis and favoring a resorptive phenotype (Ren et al., 2022). The combined effect is a progressive imbalance in which osteoblast-mediated bone formation is suppressed while osteoclast-mediated bone degradation is accelerated. This imbalance results in net peri-implant bone loss and weakens the structural foundation required for long-term implant stability. These pathological changes have direct clinical consequences. Stable osseointegration depends on intimate contact and functional integration between the implant surface and surrounding bone (Insua et al., 2024). When peri-implant bone is progressively resorbed or fails to regenerate, the implant–bone interface becomes mechanically vulnerable. Micro-motion, reduced load transfer, and loss of structural support may then further aggravate local inflammation and bone destruction, increasing the risk of implant loosening and mechanical failure (Kohli et al., 2021). Therefore, the skeletal consequences of PJI are not merely secondary damage caused by infection; they are central determinants of treatment outcome and implant survival.

Importantly, even when antimicrobial therapy or surgical intervention reduces bacterial burden, persistent impairment of bone regeneration may continue to compromise clinical recovery. In such cases, infection control alone does not necessarily restore a regenerative microenvironment. Residual inflammation, oxidative stress, MSC dysfunction, osteoblast suppression, and osteoclast overactivation may continue to prevent bone homeostasis from being re-established. These observations underscore that effective PJI treatment must address both sides of the pathological process: eradication or suppression of infection and restoration of the regenerative capacity of the peri-implant bone microenvironment. From this perspective, therapeutic strategies capable of simultaneously modulating inflammation, protecting osteogenic cells, restraining pathological bone resorption, and promoting osseointegration are essential for achieving durable reconstruction of the implant–bone interface.

### 2.4 PJI as an implant-driven osteoimmune disorder

A defining feature of PJI is its localization at the implant–tissue interface. Unlike conventional osteomyelitis, where infection is distributed within bone tissue, PJI is centered around a biomaterial surface that continuously influences both bacterial behavior and host responses. This interface therefore represents a critical nexus where biofilm formation, immune activation, and mechanical integration converge (Sauer et al., 2022). The presence of the implant introduces unique challenges. It provides a stable substrate for biofilm persistence, alters local mechanical and biological conditions, and limits immune accessibility (Ciofu et al., 2022). Consequently, infection and regeneration processes become tightly coupled at the interface. This coupling distinguishes PJI from other bone infections and necessitates specialized therapeutic strategies. From this perspective, PJI can be conceptualized as a biofilm-driven osteoimmune disorder occurring at a biomaterial interface. This conceptual shift has important implications for biomaterial design. Rather than focusing solely on antimicrobial activity, effective interventions must address the interface as a dynamic biological system, integrating biofilm control, immune modulation, and tissue regeneration (Arciola et al., 2018).

**FIGURE 1:**
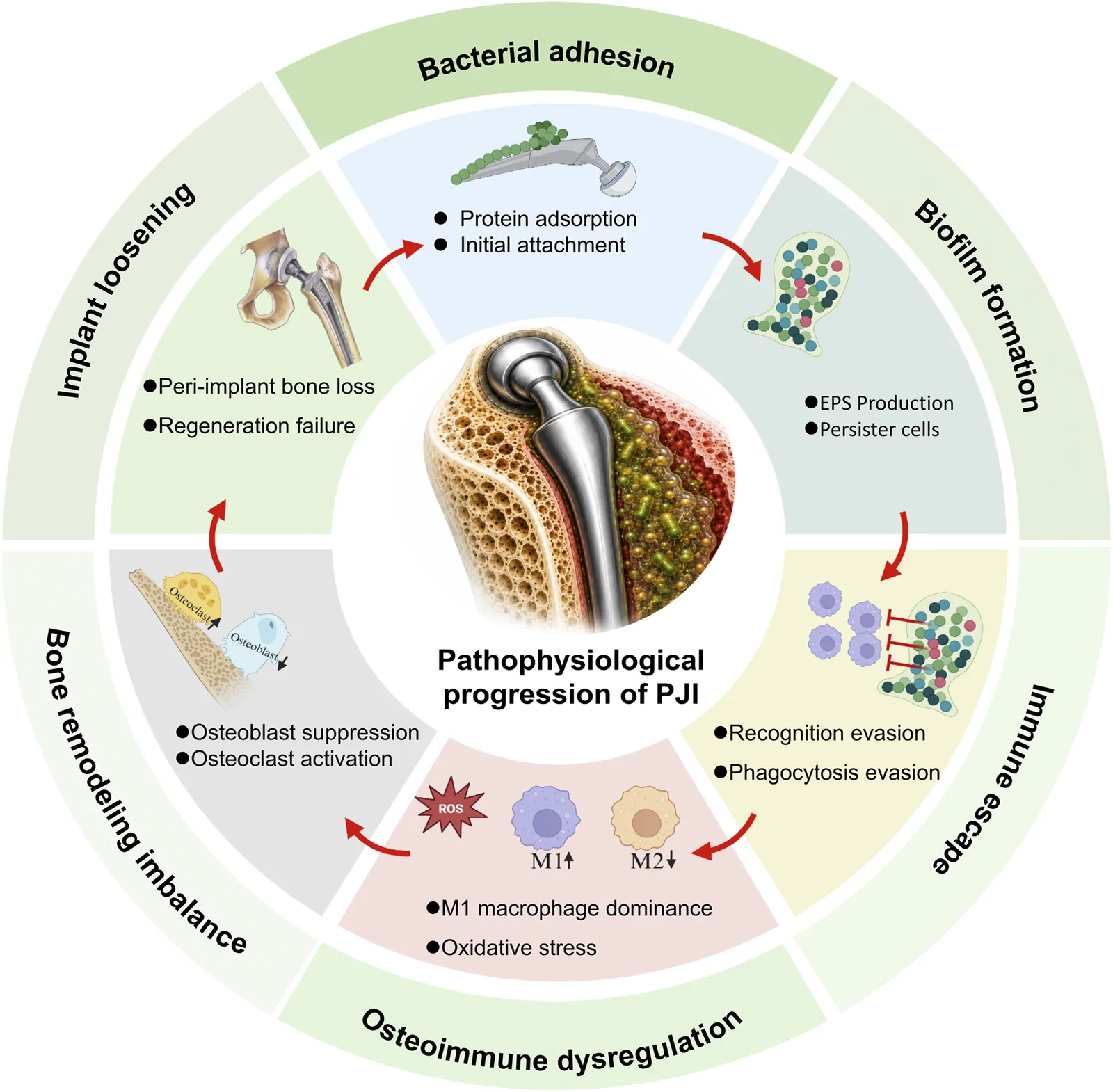
Pathophysiological progression of PJI. Bacterial adhesion initiates biofilm formation at the implant surface, followed by immune escape, persistent osteoimmune dysregulation, oxidative stress, bone-remodeling imbalance, and progressive implant loosening. The circular arrangement emphasizes the self-reinforcing nature of the implant-associated pathological cascade. Created with BioRender.com.

Based on the above mechanisms, the pathophysiological progression of PJI can be summarized as a self-reinforcing cycle involving biofilm formation, immune dysregulation, and impaired bone regeneration (Figure 1). Correspondingly, hydrogel-based strategies are increasingly designed to target multiple pathological processes simultaneously, including bacterial adhesion, biofilm persistence, oxidative stress, osteoimmune imbalance, and failed osseointegration.

## 3 Hydrogel-specific design principles for PJI

**FIGURE 2:**
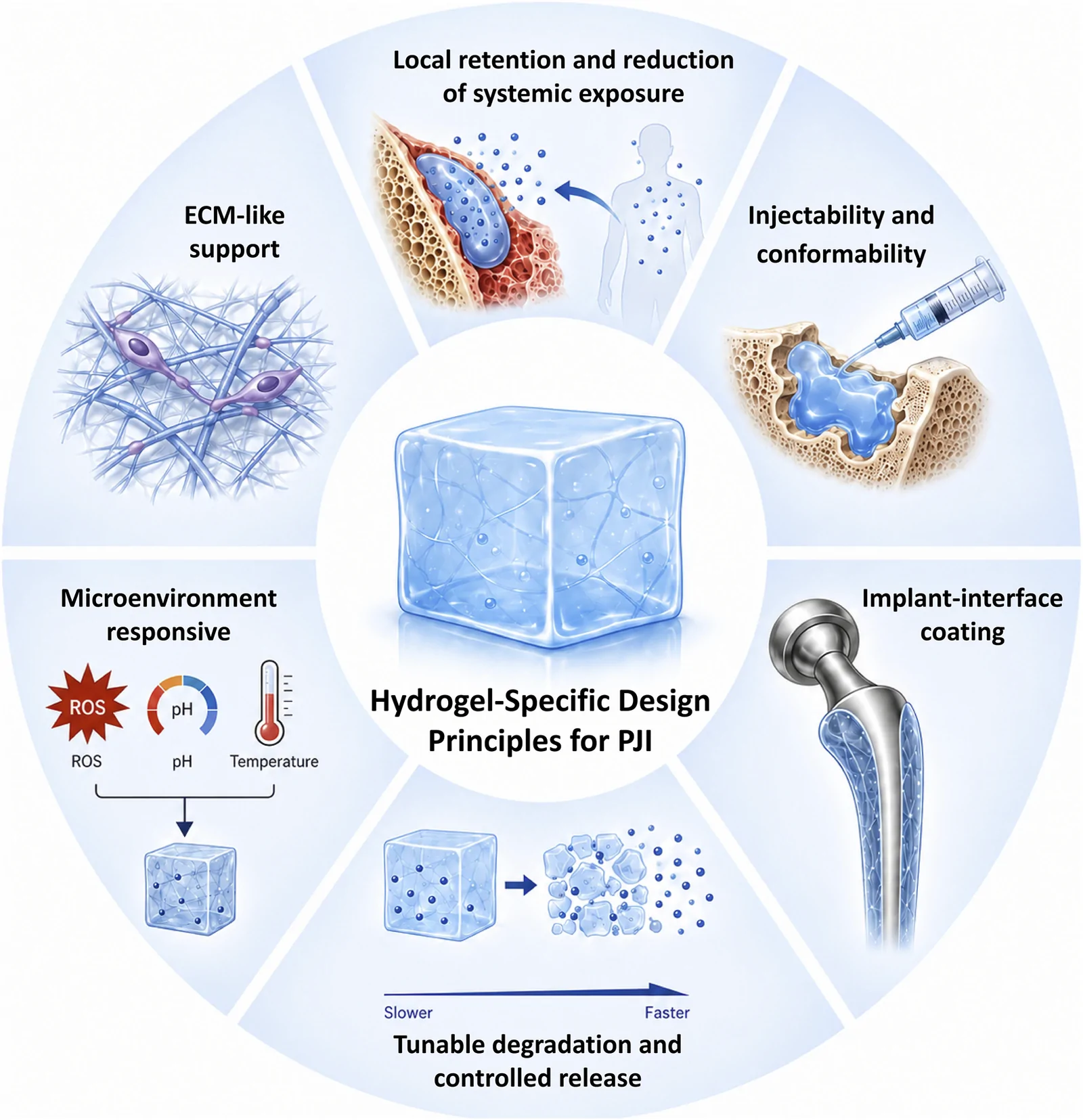
Hydrogel-specific design principles for PJI. Hydrogels provide local retention with reduced systemic exposure, injectability and conformity to irregular peri-implant defects, implant-interface coating, tunable degradation and controlled release, responsiveness to pathological microenvironmental cues, and extracellular matrix-like support. These material-level properties define the principal rationale for selecting hydrogels in appropriate PJI scenarios.

PJI presents a distinctive biomaterial and biological environment that differs from a conventional wound or isolated bone defect. A local platform must function at a wet metal-tissue interface, fill irregular dead space after debridement, resist washout, and coordinate infection control with later repair. Hydrogels are useful when their physical and chemical design directly addresses these requirements; the following subsections define those hydrogel-specific contributions and their limitations. These hydrogel-specific advantages are summarized in Figure 2.

### 3.1 Local retention and reduction of systemic exposure

A major rationale for local biomaterial therapy in PJI is the difficulty of achieving sustained antimicrobial concentrations at the implant–tissue interface through systemic administration alone (Visperas et al., 2022; Aparicio-Blanco et al., 2025). Systemically administered antibiotics are distributed throughout the body, while drug penetration into poorly vascularized peri-implant spaces and mature biofilms may remain limited. Increasing the systemic dose may improve tissue exposure but also raises the risk of nephrotoxicity, ototoxicity, gastrointestinal complications, and disruption of the normal microbiota. These limitations are particularly relevant during prolonged treatment courses or when high local concentrations are required to suppress biofilm-associated organisms.

Hydrogels can increase local retention by physically confining therapeutic agents within a crosslinked polymer network (Hameed et al., 2024; Andresen et al., 2026). After implantation or *in situ* gelation, the material forms a localized depot from which the incorporated payload diffuses or is released during network degradation. This strategy can reduce rapid clearance by blood, lymphatic drainage, or wound exudate and may maintain local concentrations above the minimum inhibitory or biofilm-eradicating threshold for longer periods. The hydrogel does not inherently improve the antibacterial potency of an antibiotic, but it can alter its pharmacokinetic profile by increasing residence time and limiting systemic distribution (Andresen et al., 2026). Local retention is also valuable for biologically unstable agents. AMPs, enzymes, cytokines, nucleic acids, and extracellular vesicles may undergo rapid degradation or loss of activity when administered in free form (Liu and Chen, 2024). Encapsulation within a hydrogel can reduce exposure to proteases, stabilize the payload, and decrease burst diffusion. Similarly, potentially cytotoxic metal ions, photosensitizers, or catalytic nanoparticles can be spatially confined to the pathological site, which may reduce exposure of distant tissues (Copling et al., 2023; Liu et al., 2024).

Nevertheless, local delivery does not automatically guarantee safe or effective dosing. Excessive drug accumulation may damage osteoblasts, MSCs, endothelial cells, or local immune cells (Argyrakis et al., 2026). A hydrogel with poor release control may produce an initial burst followed by prolonged subtherapeutic exposure, potentially increasing toxicity during the early phase while creating conditions favorable for bacterial persistence or antimicrobial resistance later. Local concentration should therefore be assessed together with spatial distribution, release kinetics, cellular tolerance, and the duration of therapeutic exposure.

In PJI, the optimal retention period is likely to depend on the clinical indication. A short-lived coating may be sufficient for prophylaxis during primary or revision arthroplasty, whereas treatment of established infection may require longer retention and deeper penetration into peri-implant tissues. Local persistence must also be balanced against degradation and clearance because materials that remain after their therapeutic function has ended may interfere with tissue integration or increase the risk of prolonged foreign-body persistence.

### 3.2 Injectability and conformability to irregular peri-implant spaces

PJI frequently involves anatomically complex defects. Surgical debridement may create irregular cavities around the prosthesis, within cancellous bone, or between the implant and surrounding soft tissue (Aparicio-Blanco et al., 2025). These spaces are difficult to treat using rigid or prefabricated materials because their geometry varies among patients and may change during surgery. Injectable hydrogels offer a practical advantage by flowing before gelation and subsequently conforming to the shape of the treated space. In situ-forming systems can be delivered through a syringe or catheter and may fill narrow peri-implant gaps that are inaccessible to conventional scaffolds (Liu et al., 2024). This property is potentially relevant to DAIR, where the prosthesis remains in place and the available space for local material placement is limited. Injectable hydrogels may also be used during one-stage or two-stage revision to fill residual dead space, coat exposed implant surfaces, or deliver therapeutic agents around reconstructed bone defects. Conformability can improve contact between the material and the pathological interface. A hydrogel that closely follows the contours of bone, soft tissue, and metal surfaces may reduce untreated voids and improve spatial distribution of the incorporated payload. This is particularly important for biofilm-associated infection because antimicrobial efficacy depends not only on the total administered dose but also on whether the active agent reaches colonized implant regions.

The advantages of injectability must be balanced against gelation behavior. A formulation that gels too slowly may be diluted or washed away by blood, irrigation fluid, and wound exudate. Conversely, overly rapid gelation may hinder injectability, limit uniform distribution within the defect, and reduce effective contact with the implant–tissue interface (Hameed et al., 2024; Liu et al., 2024). Gelation should therefore occur within a clinically practical window that allows preparation, application, and adjustment before wound closure. Injectability also depends on viscosity, shear-thinning behavior, polymer concentration, and needle diameter. Highly concentrated formulations may improve retention and mechanical stability but require greater injection force and may distribute unevenly. Lower-viscosity systems are easier to deliver but may leak from the target site before crosslinking. These considerations are rarely evaluated in standard *in vitro* studies but are central to surgical usability.

Another limitation is that most injectable hydrogels provide minimal load-bearing support. Their ability to fill a defect should not be equated with restoration of mechanical stability. In extensive peri-implant bone loss, injectable hydrogels may need to be combined with porous metals, calcium phosphate ceramics, bone grafts, structural spacers, or other mechanically robust materials.

### 3.3 Implant-interface coating and wet-surface adaptability

The implant surface is the primary site of bacterial adhesion and early biofilm formation. Following implantation, host proteins rapidly adsorb onto the material and create a conditioning film that can support both bacterial attachment and host-cell integration. This competition is often described as a “race for the surface,” in which early bacterial colonization may prevent effective tissue integration and establish a persistent biofilm niche (Kalelkar et al., 2021; Bohara and Suthakorn, 2022). Hydrogel coatings are designed to intervene directly at this critical interface. A coating-type hydrogel can provide several functions. It may create a temporary physical barrier that reduces bacterial contact with the implant, present anti-fouling or anti-adhesive chemical groups, and retain locally administered antibiotics or other antibacterial agents (Wang et al., 2023). By restricting these agents to the implant surface, the coating can produce high local exposure during the early postoperative period without requiring high systemic doses. The DAC, for example, illustrates the clinical feasibility of applying a resorbable antibiotic-loaded hydrogel to an implant during surgery (Bove et al., 2025).

Surface adaptation is particularly challenging under operative conditions because implants are exposed to blood, saline, proteins, and tissue fluids. Hydrogels intended for intraoperative coating must therefore adhere to wet surfaces or form stable conformal layers despite continuous fluid exposure. Catechol-containing groups, dynamic covalent bonds, electrostatic interactions, and interpenetrating polymer networks have been investigated to improve wet adhesion (Yuk et al., 2022). Surface chemistry must nevertheless remain compatible with osteoblast attachment and osseointegration, particularly when the coating is applied to porous or bioactive implant regions.

Coating thickness and degradation are also important. A thin coating may preserve implant geometry and surface roughness but have limited loading capacity. A thick coating may retain more therapeutic material but could fill pores, alter friction during implant insertion, or delay direct bone contact (Wang et al., 2023). The degradation period should be long enough to cover the highest-risk phase of bacterial adhesion but short enough to avoid impairing long-term tissue integration.

Hydrogel coatings are likely to be most effective for prevention or very early infection (Bove et al., 2025). They have limited capacity to treat mature biofilms extending into surrounding bone and soft tissue, particularly after the coating has degraded (Roberts et al., 2025). Thus, interface coatings should be viewed as prophylactic or adjunctive technologies rather than universal solutions for chronic PJI. Their clinical value depends on the timing of application, bacterial burden, debridement quality, payload selection, and compatibility with the implant fixation strategy.

### 3.4 Tunable degradation and controlled release

The polymer network of a hydrogel determines the movement of water, solutes, and therapeutic agents. Release can occur through diffusion, swelling, reversible binding, hydrolysis, enzymatic cleavage, or bulk and surface degradation. By modifying polymer molecular weight, crosslinking density, mesh size, hydrophilicity, and degradable linkages, investigators can adjust the duration and pattern of payload release (Hameed et al., 2024; Andresen et al., 2026). This tunability is especially relevant to PJI because the required treatment period differs among clinical settings. Prophylactic application may require high antimicrobial exposure during the first postoperative days, whereas established infection may require sustained delivery over weeks. Immunomodulatory or regenerative agents may need to be introduced only after bacterial burden has been reduced (Liu et al., 2024). A single release profile is therefore unlikely to be appropriate for every stage of disease.

Degradation-controlled release offers another strategy. Hydrolytically or enzymatically cleavable networks can release their payload as the polymer structure breaks down. Ideally, degradation should match the duration of infection control and tissue repair (Hameed et al., 2024). Premature degradation can cause loss of local retention and release a large fraction of the payload before it is required. Excessively slow degradation can leave residual material that occupies space, delays tissue infiltration, or provokes a foreign-body response. The biological properties of degradation products must also be considered. Breakdown fragments may alter local pH, osmolarity, inflammation, or cellular metabolism. In infection, acidic degradation products could intensify an already acidic microenvironment, whereas rapid release of polymer fragments may alter macrophage or neutrophil activity. Biocompatibility testing should therefore evaluate both the intact hydrogel and its products over clinically relevant time periods.

Controlled release should not be defined simply as prolonged release. An effective system must maintain therapeutic concentrations within an appropriate range, avoid early toxicity, prevent late subtherapeutic exposure, and coordinate release with disease progression (Andresen et al., 2026). This requires quantitative evaluation under conditions that approximate peri-implant fluid flow, protein adsorption, bacterial colonization, and mechanical loading rather than relying exclusively on static buffer-based release studies.

### 3.5 Microenvironment-responsive and sequential delivery

Compared with healthy tissue, the infected peri-implant microenvironment exhibits a series of characteristic pathological changes, including local acidification, excessive ROS generation, increased proteolytic and bacterial enzyme activity, hypoxia, and sustained inflammatory signaling (Brandquist and Kielian, 2025). Stimuli-responsive hydrogels incorporate chemical bonds or structural components that change in response to these pathological cues. Such systems aim to restrict therapeutic activation to infected regions and to increase delivery when pathological activity is greatest.

pH-responsive hydrogels may swell, degrade, or alter drug binding under acidic conditions generated by bacterial metabolism and inflammatory tissue injury (Thambi et al., 2023). ROS-responsive systems use oxidation-sensitive linkages, such as thioketal, boronic ester, or related groups, to trigger degradation or release in oxidative environments (Pu et al., 2024). Enzyme-responsive hydrogels can be designed to react to bacterial enzymes, matrix metalloproteinases, or other proteases enriched at infected sites (Sobczak, 2022). External triggers, including near-infrared (NIR) light, ultrasound, electric fields, and magnetic fields, offer additional control but require suitable equipment and adequate penetration into deep peri-articular tissues.

Responsive release has two potential advantages. First, it can reduce unnecessary exposure in relatively healthy tissue while increasing treatment intensity at sites with active infection or inflammation. Second, it can couple therapeutic delivery to temporal changes in the disease. However, responsiveness is only useful if the chosen stimulus is sufficiently specific, predictable, and reproducible. Acidity and ROS are not unique to infection and also occur during sterile inflammation and tissue repair. Interpatient variability in pH, oxidative stress, bacterial species, and enzyme expression may lead to inconsistent activation. Responsive systems should therefore be validated across clinically relevant ranges rather than at a single experimentally convenient stimulus level.

Sequential therapy is a particularly important design goal because successful bone healing follows a temporally ordered inflammatory-to-regenerative sequence (Duda et al., 2023). Early PJI management prioritizes bacterial killing and biofilm disruption, whereas later repair requires inflammatory resolution, angiogenesis, osteogenesis, and balanced bone remodeling. Simultaneous release of all components may be counterproductive. Strong oxidative or photothermal antibacterial treatment can damage MSCs and osteoblasts, while premature delivery of growth factors may be ineffective in the presence of active infection and excessive inflammation. Hydrogels can potentially separate these phases through multilayer structures, differential binding affinities, degradable compartments, nanoparticles embedded within the gel, or stimulus-dependent switching. An initial phase may release antibiotics, AMPs, enzymes, or photothermal agents, followed by a later phase that delivers antioxidants, immunomodulatory molecules, angiogenic factors, or osteogenic cues. Such stage-adaptive systems more closely reflect the biological sequence required for successful healing (Deng et al., 2024; Qiao et al., 2024).

Despite their conceptual appeal, responsive and sequential hydrogels are among the most complex systems to translate. Multiple components increase manufacturing variability, sterilization sensitivity, storage requirements, regulatory burden, and the difficulty of defining dose and release specifications. Each added function should therefore address a clinically meaningful limitation rather than simply increase material complexity.

### 3.6 ECM-like support and integration with mechanically robust materials

Hydrogels contain large amounts of water and can reproduce selected biochemical and structural features of the native ECM. Their polymer networks may present cell-adhesive ligands, protease-sensitive sequences, growth-factor-binding domains, and adjustable mechanical cues. These properties can support cell adhesion, migration, proliferation, and differentiation and may help reconstruct a local environment favorable to bone and vascular regeneration after infection control (Lu P. et al., 2024).

The ECM-like function of a hydrogel should not be overstated. A hydrated polymer network may provide a permissive cellular microenvironment, but it does not reproduce the full biochemical, hierarchical, and mechanical complexity of peri-implant bone (Shapiro and Oyen, 2016). Large bone defects require mineralized matrix formation, vascular invasion, load transfer, and long-term remodeling. Most hydrogels have substantially lower compressive strength, fatigue resistance, and fracture toughness than cortical or trabecular bone and cannot independently stabilize a mechanically compromised implant (Yu et al., 2022). Integration with rigid materials may therefore be necessary. Hydrogels can be combined with calcium phosphate ceramics, bioactive glass, porous titanium, three-dimensional printed scaffolds, bone grafts, or structural spacers. In such composites, the rigid component provides mechanical support and osteoconductive architecture, while the hydrogel contributes local retention, biological payload delivery, cell support, and interface filling. Nanoparticles, microspheres, or liposomes can also be incorporated into the hydrogel to improve loading, protect unstable agents, or introduce responsive release.

Ultimately, ECM-like support is most valuable when integrated with infection control and mechanical reconstruction. In PJI, regenerative performance cannot be assessed only through osteogenic marker expression under ideal culture conditions. Relevant evaluation should include bacterial clearance, inflammatory resolution, vascularization, bone–implant contact, implant stability, and restoration of load-bearing function.

## 4 Sources of biological activity in hydrogel-based systems

Although hydrogels have emerged as versatile platforms for the local treatment of PJI, the biological origin of their therapeutic effects is often interpreted ambiguously. In many studies, antibacterial, antibiofilm, antioxidative, immunomodulatory, angiogenic, and osteogenic activities are broadly attributed to “hydrogel-based systems.” However, these biological functions frequently arise from different sources. Some are intrinsic to the hydrogel matrix itself, whereas others are primarily mediated by incorporated antibiotics, AMPs, nanoparticles, enzymes, growth factors, or cells. In addition, hydrogels may enhance therapeutic efficacy through material-specific properties, including local retention, controlled release, interface adaptation, and microenvironment-responsive delivery, even when they possess little intrinsic biological activity.

**FIGURE 3:**
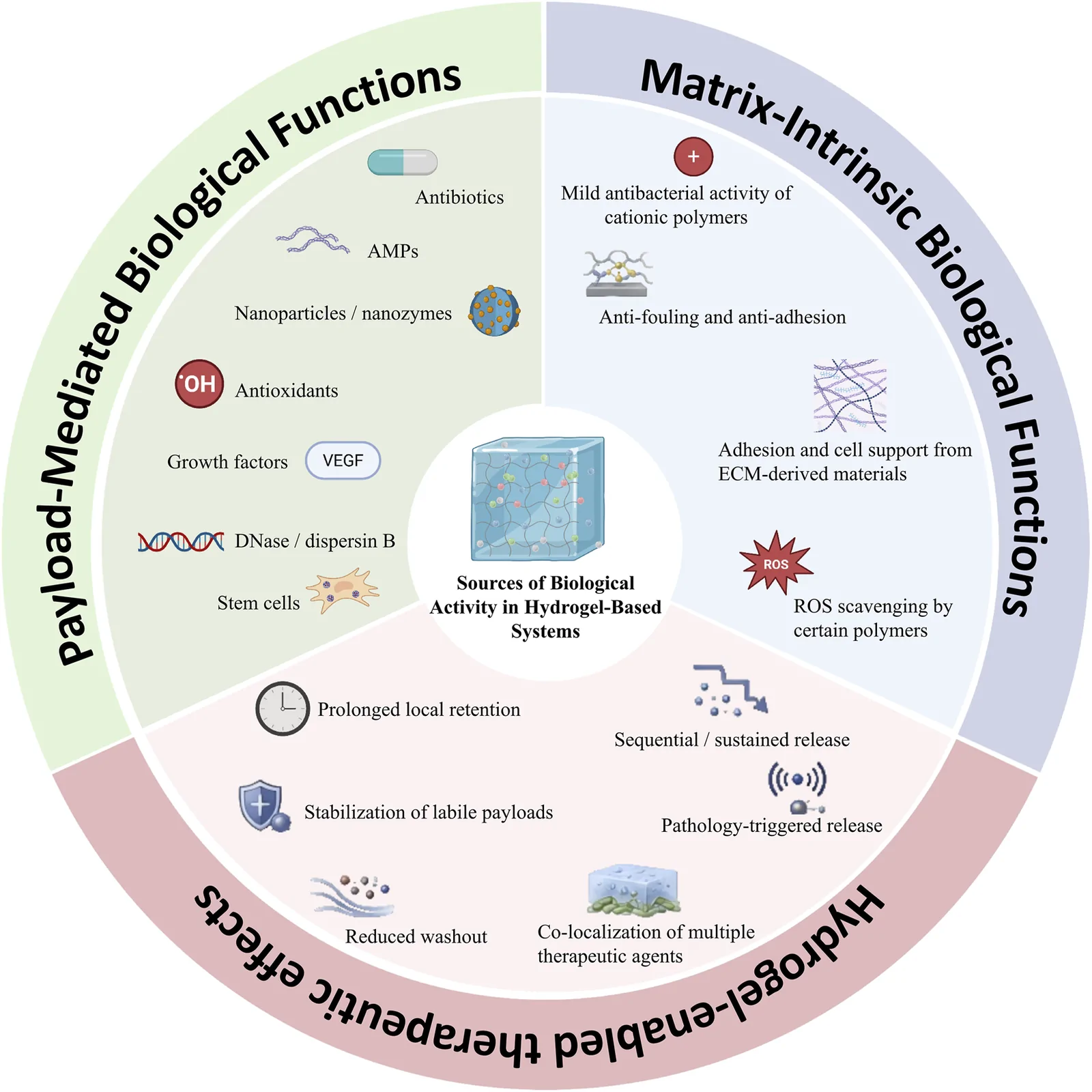
Sources of biological activity in hydrogel-based systems. Matrix-intrinsic biological functions arise from the polymer network, payload-mediated biological functions arise from incorporated therapeutic agents, and hydrogel-enabled delivery effects arise from local retention, payload protection, conformity to irregular spaces, controlled release, and spatial confinement. Correct attribution of these three contributions is essential for indication-specific design and meaningful comparison among hydrogel formulations. Created with BioRender.com.

Failure to distinguish these different sources may overestimate the contribution of the hydrogel platform or obscure the actual mechanism responsible for therapeutic efficacy. Such distinction is particularly important when comparing different hydrogel formulations or evaluating their translational potential. Therefore, hydrogel-based therapies should be interpreted from three complementary perspectives: (i) matrix-intrinsic biological functions, (ii) payload-derived biological functions, and (iii) hydrogel-enabled effects. Figure 3 summarizes this attribution framework.

### 4.1 Matrix-intrinsic biological functions

Certain hydrogels possess inherent biological activity that originates from the polymer network rather than from incorporated therapeutic agents. These intrinsic functions depend primarily on polymer composition, chemical functional groups, surface properties, and degradation behavior. For example, cationic polymers such as chitosan can interact electrostatically with negatively charged bacterial membranes, resulting in membrane disruption and mild antibacterial activity (Mirbagheri et al., 2024). Hyaluronic acid, collagen, gelatin, and other ECM-derived polymers provide cell-adhesive motifs that facilitate cell migration, proliferation, and tissue remodeling (Kim et al., 2020). Antioxidant-functionalized matrices may directly scavenge ROS, whereas ROS-responsive polymers primarily respond to oxidative cues to regulate degradation or payload release. (Pu et al., 2024). Similarly, anti-fouling polymers can reduce nonspecific protein adsorption and bacterial adhesion, thereby delaying early biofilm formation on implant surfaces (Hassani et al., 2024). These intrinsic activities, however, are generally moderate compared with those achieved by dedicated antibacterial or regenerative agents. In most cases, the hydrogel matrix alone is insufficient to eradicate mature biofilms or fully restore the osteoimmune microenvironment. Therefore, intrinsic biological functions should be regarded as complementary properties that improve the local microenvironment rather than as standalone therapeutic mechanisms.

### 4.2 Payload-derived biological functions

In contrast, many biological effects reported for hydrogel-based systems originate primarily from incorporated therapeutic agents rather than from the hydrogel itself. Antibiotics remain the principal source of bactericidal activity, while AMPs disrupt bacterial membranes through membrane permeabilization (Hancock et al., 2021; Andresen et al., 2026). DNase, dispersin B, and other enzymes degrade EPS to facilitate biofilm disruption (Flemming et al., 2023). Metal ions, nanozymes, and photothermal or photodynamic agents provide antibacterial activity through oxidative stress, membrane damage, or localized thermal effects (Kalelkar et al., 2021). Likewise, growth factors, cytokines, small-molecule drugs, stem cells, and extracellular vesicles are primarily responsible for immunomodulation, angiogenesis, osteogenesis, and tissue regeneration (Kang et al., 2021). In these systems, the hydrogel should not be considered the direct source of biological activity. Instead, it functions predominantly as a local delivery matrix that retains, protects, and gradually releases the active payload. Consequently, the observed therapeutic efficacy should be attributed primarily to the incorporated agents, while recognizing the essential role of the hydrogel in optimizing their spatial and temporal delivery.

### 4.3 Hydrogel-enabled therapeutic effects

Beyond intrinsic matrix activity and payload-derived functions, hydrogels provide a third level of therapeutic benefit through material-enabled synergy. Although the hydrogel may not directly kill bacteria or stimulate tissue regeneration, its physicochemical properties can substantially enhance the performance of incorporated therapeutic agents. First, hydrogels prolong local retention at the implant–bone interface, reducing rapid clearance by blood flow or wound exudate and maintaining therapeutic concentrations at the infection site (Andresen et al., 2026). Second, the polymer network protects unstable payloads such as AMPs, proteins, nucleic acids, and extracellular vesicles from premature degradation (Lu P. et al., 2024). Third, controlled degradation and diffusion enable sustained or sequential release, allowing antibacterial therapy to dominate the early infection phase while immunomodulatory or regenerative signals are delivered during later stages of healing (Qiao et al., 2024). Fourth, injectable and conformable hydrogels closely adapt to irregular peri-implant spaces, maximizing contact with biofilms and surrounding tissues (Aparicio-Blanco et al., 2025). Finally, stimuli-responsive hydrogels can release therapeutic agents in response to pathological signals, including acidic pH, elevated ROS levels, bacterial enzymes, or external physical stimuli, thereby improving therapeutic specificity while reducing unnecessary systemic exposure (Liu et al., 2024). These hydrogel-based properties represent the principal material-level advantages of hydrogels over conventional local delivery platforms. Importantly, they arise from the structural and physicochemical characteristics of the hydrogel matrix rather than from the biological activity of individual payloads.

## 5 Hydrogel-based strategies for infection control and biofilm disruption

Effective treatment of PJI requires not only bacterial eradication but also disruption of mature biofilms that protect microorganisms from host immunity and antimicrobial therapy. Although antibacterial activity is primarily mediated by incorporated antibiotics, AMPs, enzymes, or nanoparticles, hydrogels significantly enhance therapeutic performance by improving local retention, protecting unstable payloads, enabling controlled or stimuli-responsive release, and adapting to the complex implant–tissue interface. Therefore, the therapeutic value of hydrogels lies in their ability to optimize the spatial and temporal delivery of anti-infective agents rather than serving as the primary source of antibacterial activity. The following sections discuss representative hydrogel-based strategies for infection control while distinguishing the respective contributions of the hydrogel matrix and the incorporated therapeutic payloads. Evidence relevance is identified explicitly, and representative systems are summarized in Table 1.

**TABLE 1:** Representative hydrogel systems evaluated in PJI and PJI-relevant orthopedic infection models.

Therapeutic strategy/representative study	Hydrogel matrix/payload	Primary source of biological activity	Hydrogel-specific contribution	Release or activation	Experimental model/PJI evidence level	Main implication	Key translational limitation
Local antibiotic delivery Boot et al. (2022)	Thermoresponsive hyaluronic acid hydrogel; gentamicin + vancomycin	Payload-derived	Injectable local depot; conformability; high local retention; reduced systemic exposure	Diffusion and hydrogel dissolution	Sheep orthopedic device-related infection; prophylaxis and revision treatment Implant-associated large-animal model	Strong evidence that local hydrogel delivery can augment systemic antimicrobial therapy	Antibiotic-dependent; not an arthroplasty PJI trial; limited animal numbers
Local antibiotic coating De Meo et al. (2023)	DAC hydrogel (hyaluronic acid + poly-lactic acid); surgeon-selected antibiotics	Payload-derived	Temporary implant coating and peri-implant drug localization	Resorption with local antibiotic diffusion	Acute clinical PJI treated with DAIR Clinical PJI	Demonstrates intraoperative feasibility in a true PJI treatment pathway	Preliminary, small nonrandomized comparison; most applicable to early infection
Antimicrobial peptide delivery Feng et al. (2022)	Thermosensitive peptide hydrogel; antimicrobial peptides (AMPs)	Payload-derived	Protects AMPs from degradation and prolongs local exposure	Thermally induced gelation with sustained peptide release	MRSA-infected wound Non-implant infection	Supports hydrogel-enabled stabilization and local delivery of AMPs	Wound model; no implant, biofilm-interface, or PJI validation
Biofilm disruption Lin et al. (2024)	Porous ternary AuAgCu hydrogel nanozyme with immobilized DNase	Payload-derived + matrix/catalytic activity	Prolonged enzyme–biofilm contact; enzyme stabilization; combined matrix disruption and catalytic killing	Immobilized DNase activity plus nanozyme-mediated antibacterial action	MRSA infection model Non-implant infection	Illustrates hydrogel-enabled antibiofilm combination therapy	Metal safety, sterilization, and dose control require validation; no PJI model
ROS-responsive antibacterial therapy Qiao et al. (2024)	HA-PBA/PVA hydrogel; moxifloxacin and curcumin-loaded pluronic micelles	Payload-derived + hydrogel-enabled	ROS-responsive sequential delivery of antibacterial and anti-inflammatory therapy	Phenylboronate ester cleavage in ROS-rich conditions	MRSA-infected mouse skin wound Non-implant infection	Demonstrates temporal separation of infection control and inflammation resolution	Stimulus variability and wound-model extrapolation limit direct PJI claims
pH-responsive therapy Delshad Chermahini et al. (2026)	HA/pβae hydrogel; therapeutic enzymes	Payload-derived + hydrogel-enabled	Acidity-responsive release and inflammation-adaptive delivery	pH-dependent network response and enzyme release	Infected wound Non-implant infection	Shows how inflammatory acidity can be used to adapt local therapeutic release	No implant-associated or PJI validation; clinical stimulus ranges remain uncertain
Immunoregulatory therapy Wang et al. (2024)	Injectable immunoregulatory hydrogel; immunomodulatory molecules	Payload-derived + hydrogel-enabled	Localized delivery and sequential regulation of macrophage polarization	Sequential or sustained payload release	Infected wound Non-implant infection	Supports temporal coordination of antibacterial treatment and immune resolution	Evidence derived from a wound model; macrophage responses in PJI remain unvalidated
Redox regulation Sun et al. (2023)	Bone-microenvironment-regulating hydrogel; ROS-scavenging and oxygen-generating components	Matrix-intrinsic + payload-derived	Prolonged ROS scavenging and sustained oxygen generation	Sustained redox regulation within the hydrogel network	Bone defect Non-infected/non-implant model	Demonstrates hydrogel-supported regulation of oxidative stress and oxygen availability	No infection, biofilm, implant, or PJI validation
Angiogenic–osteogenic delivery Zhang et al. (2022)	Self-assembled peptide hydrogel; VEGF + BMP-2	Payload-derived	Sustained co-delivery of angiogenic and osteogenic factors	Diffusion and network degradation	Bone regeneration Non-infected model	Supports coordinated angiogenic and osteogenic factor delivery	No infection, implant interface, or PJI validation
ECM-mimetic regenerative delivery Man et al. (2022)	Chitosan–collagen ECM-mimetic hydrogel; osteoblast-derived extracellular vesicles	Matrix-intrinsic + payload-derived	ECM-like support; enhanced cell adhesion; controlled extracellular-vesicle retention	Diffusion and network degradation	*In vitro* bone regeneration model Non-infected regeneration	Clarifies the hydrogel contribution to regenerative payload delivery	No infection, biofilm, implant, or *in vivo* PJI validation
Anti-osteolytic therapy Li et al. (2026)	Bisphosphonate hydrogel; bisphosphonate	Payload-derived	Localized anti-resorptive delivery with prolonged retention	Sustained local release from the hydrogel network	Fracture-related infection Implant-related orthopedic infection, not PJI	Links infection control with preservation of cortical bone and suppression of pathological resorption	FRI model; direct applicability to arthroplasty PJI remains uncertain
Neuro-osteoimmune regeneration Jing et al. (2023)	GelMA hydrogel containing magnesium-modified black phosphorus (BP@Mg)	Matrix-intrinsic + hydrogel-enabled	Local retention, photothermal activation, and a conductive microenvironment	Near-infrared activation and material degradation	Infected calvarial bone-defect model Infected bone defect, not PJI	Links antibacterial treatment with neural and bone regeneration	External activation, skull model, and no prosthetic interface

### 5.1 Local antibiotic delivery

Local antibiotic delivery represents the most established hydrogel-based strategy for PJI management. The antibacterial effect is primarily provided by the incorporated antibiotic, whereas the hydrogel functions as a localized delivery platform that improves drug retention, regulates release kinetics, and reduces systemic exposure (Andresen et al., 2026). Compared with systemic administration, hydrogel-based delivery can maintain higher antibiotic concentrations at the implant–tissue interface, where bacterial biofilms are most difficult to eradicate (Boot et al., 2022). The therapeutic advantage of hydrogels lies in their material properties rather than their intrinsic antibacterial activity. Injectable hydrogels can fill irregular peri-implant spaces following debridement, while coating hydrogels can deliver antibiotics directly to implant surfaces during the early stages of bacterial colonization (Aparicio-Blanco et al., 2025). Furthermore, tunable crosslinking and degradation enable sustained antibiotic release, prolonging local exposure and reducing rapid drug clearance. These characteristics are particularly valuable for preventing early biofilm establishment and enhancing local antimicrobial efficacy.

Despite their clinical promise, antibiotic-loaded hydrogels are unlikely to overcome the limitations of antibiotic therapy on their own. Their efficacy remains constrained by bacterial susceptibility and declines against mature biofilms, persister cells, and multidrug-resistant organisms. In addition, poorly controlled release may result in transient overdosing followed by prolonged subtherapeutic exposure, creating conditions that favor resistance selection. Future progress will depend less on increasing antibiotic loading and more on integrating optimized release profiles with complementary antibiofilm and host-directed therapeutic strategies.

### 5.2 Delivery of AMPs

AMPs have emerged as promising alternatives to conventional antibiotics because of their broad-spectrum antibacterial activity, rapid membrane-disruptive mechanisms, and relatively low propensity to induce bacterial resistance. Unlike traditional antibiotics that often target specific metabolic pathways, AMPs primarily disrupt bacterial membrane integrity, making them effective against multidrug-resistant pathogens and, in some cases, biofilm-associated bacteria (Hancock et al., 2021). However, their clinical application is limited by poor stability, rapid enzymatic degradation, short local residence time, and potential cytotoxicity at high concentrations. Hydrogels provide an effective platform for overcoming these limitations by improving the local delivery of AMPs rather than enhancing their intrinsic antibacterial activity. Encapsulation within a hydrogel matrix protects AMPs from proteolytic degradation, prolongs their retention at the implant–tissue interface, and enables sustained release over time. Injectable hydrogels can further distribute AMPs throughout irregular peri-implant spaces, increasing their contact with biofilm-colonized surfaces while reducing systemic exposure (Feng et al., 2022). These material-specific advantages help maintain therapeutic peptide concentrations within infected tissues and improve overall treatment efficiency.

Although AMP-loaded hydrogels have shown encouraging therapeutic potential, several challenges remain before clinical application. The antibacterial activity of AMPs can be influenced by the local microenvironment, including ionic strength, pH, and interactions with serum proteins, while excessive peptide loading may impair the viability of surrounding host cells. Moreover, most evidence is still based on *in vitro* experiments or non-implant infection models, with relatively limited validation in clinically relevant models of implant-associated PJI. Future work should focus on improving peptide stability and release behavior while establishing more representative preclinical models to evaluate efficacy, safety, and translational potential under conditions that better reflect clinical practice.

### 5.3 Biofilm disruption

Biofilm disruption represents a critical step in PJI treatment because mature biofilms markedly reduce antimicrobial penetration and protect bacteria from host immune clearance (Flemming et al., 2023). While enzymes, biofilm-dispersing molecules, and catalytic nanoparticles are primarily responsible for degrading the EPS matrix, hydrogels enhance their therapeutic efficacy by improving local retention, sustained release, and prolonged contact with biofilm-colonized implant surfaces (Lin et al., 2024). Hydrogels are particularly advantageous for delivering biofilm-disrupting agents such as DNase I, dispersin B, proteases, and nanozymes. Their hydrated three-dimensional network enables close adaptation to irregular peri-implant spaces, allowing these agents to remain localized at the infection site and continuously interact with the biofilm matrix. In addition, controlled release can maintain enzymatic activity over extended periods, facilitating gradual EPS degradation and improving the penetration of subsequently released antibiotics or antimicrobial peptides. This sequential strategy is especially attractive for mature biofilms, where disruption of the protective matrix is often a prerequisite for effective bacterial eradication.

Despite encouraging experimental results, several challenges remain. Enzymes may lose activity during long-term storage or after sterilization, and excessive degradation of the biofilm matrix may release planktonic bacteria, potentially increasing the risk of dissemination if not combined with effective antimicrobial therapy (Rumbaugh and Sauer, 2020). Furthermore, most biofilm-disrupting hydrogels have been evaluated in vitro biofilm models or non-implant infection models, whereas evidence from clinically relevant implant-associated PJI models remains limited. Therefore, future studies should focus on integrating biofilm disruption with sustained antimicrobial delivery and validating these strategies in representative PJI models to improve their translational potential.

### 5.4 Stimuli-responsive anti-infective systems

Stimuli-responsive hydrogels represent an advanced strategy for improving the precision of local anti-infective therapy in PJI. Rather than continuously releasing therapeutic agents, these hydrogels respond to pathological cues within the infected microenvironment, such as acidic pH, elevated ROS, bacterial enzymes, or externally applied stimuli, thereby enabling on-demand drug release. Importantly, the antimicrobial activity remains dependent on the incorporated antibiotics, antimicrobial peptides, or other bioactive agents, whereas the hydrogel provides the responsive delivery mechanism. The principal advantage of stimuli-responsive hydrogels is their ability to synchronize drug release with disease progression. For example, ROS- or pH-responsive hydrogels can accelerate degradation and payload release under inflammatory conditions while remaining relatively stable in healthy tissues (Qiao et al., 2024; Delshad Chermahini et al., 2026). Similarly, enzyme-responsive systems exploit bacterial or host-derived enzymes to trigger localized drug delivery, thereby improving therapeutic specificity and reducing unnecessary drug exposure (Zhang et al., 2024). These responsive properties not only enhance local antimicrobial efficacy but also create opportunities for sequential therapy, in which early antibacterial treatment is followed by immunomodulatory or regenerative interventions as infection resolves.

Although responsive hydrogels have considerable therapeutic potential, several challenges remain before clinical translation. The pathological stimuli present in PJI are highly heterogeneous and may vary with bacterial species, infection stage, and host immune status, making it difficult to achieve predictable activation. Moreover, increasing material complexity may compromise manufacturing reproducibility, sterilization, storage stability, and regulatory approval. Therefore, future development should prioritize robust and clinically relevant responsive designs that provide clear therapeutic advantages over conventional sustained-release systems rather than pursuing increasingly sophisticated but difficult to translate multifunctional platforms.

### 5.5 Combination and sequential therapy

Given the multifactorial pathogenesis of PJI, monotherapy is often insufficient to eradicate mature biofilms and restore the peri-implant microenvironment. Hydrogels provide a versatile platform for combination therapy by co-delivering multiple therapeutic agents with complementary mechanisms of action. Rather than contributing direct antibacterial activity, the hydrogel enables spatial localization, protects different payloads, and coordinates their release to maximize synergistic therapeutic effects. Representative combination strategies include antibiotics combined with AMPs, biofilm-dispersing enzymes, metal nanoparticles, nanozymes, or immunomodulatory molecules (Lu Y. et al., 2024; Qiao et al., 2024). Such combinations can simultaneously target bacterial viability, disrupt the EPS matrix, regulate excessive inflammation, and promote subsequent tissue repair. Moreover, hydrogels can be engineered to achieve sequential release, allowing antibacterial agents to be delivered during the early stage of infection, followed by anti-inflammatory or osteogenic factors as the inflammatory burden decreases. This stage-specific delivery more closely matches the biological progression of PJI than simultaneous release of all therapeutic components.

Greater functionality often comes at the cost of greater complexity. Incorporating multiple therapeutic agents requires careful coordination of loading efficiency, release profiles, material compatibility, and storage stability, while also increasing the demands associated with manufacturing, sterilization, quality assurance, and regulatory approval. Progress toward clinical application will depend less on maximizing multifunctionality than on developing robust and reproducible systems whose added complexity translates into clear therapeutic benefit.

## 6 Hydrogel-based osteoimmune and regenerative regulation

Successful treatment of PJI requires more than bacterial eradication. Although effective antimicrobial therapy can reduce the infectious burden, restoration of the peri-implant microenvironment is equally critical for long-term implant stability and functional recovery. Persistent inflammation, excessive oxidative stress, impaired angiogenesis, dysregulated osteoclast activity, and suppressed osteogenesis often continue after infection control, preventing reestablishment of bone homeostasis and successful osseointegration (Bandick et al., 2023). Therefore, local therapeutic strategies should not only eliminate pathogens but also facilitate the transition from infection-driven inflammation to tissue regeneration. Hydrogels provide a versatile platform for coordinating this process by integrating immunomodulatory and regenerative therapies with localized delivery. Importantly, many of the biological effects discussed in this section including macrophage polarization, redox regulation, angiogenesis, and osteogenesis are primarily mediated by incorporated cytokines, small molecules, nanoparticles, growth factors, or cells rather than by the hydrogel matrix itself. The principal contribution of hydrogels lies in improving local retention, protecting bioactive payloads, enabling controlled or sequential release, and responding to changes in the peri-implant microenvironment. These material-specific properties allow therapeutic signals to be delivered at the appropriate location and stage of healing, thereby supporting the coordinated restoration of osteoimmune homeostasis.

The following sections summarize representative hydrogel-based strategies for osteoimmune regulation and tissue regeneration, with particular emphasis on the distinct roles of therapeutic payloads and hydrogel platforms, as well as their current translational potential in clinically relevant PJI.

### 6.1 Regulation of macrophage state transitions

Macrophages are central regulators of the osteoimmune microenvironment during PJI (Masters et al., 2022; Weivoda and Bradley, 2023). Following bacterial infection, macrophages initially adopt a pro-inflammatory (M1) phenotype to eliminate invading pathogens through phagocytosis and the production of ROS, nitric oxide, and pro-inflammatory cytokines. However, persistent biofilm stimulation often maintains excessive M1 activation, leading to sustained secretion of TNF-α, IL-1β, and interleukin-6 (IL-6), which amplifies inflammation, promotes osteoclastogenesis, and suppresses osteogenic differentiation (Wang and He, 2020). Therefore, successful PJI treatment requires not continuous immunosuppression but a timely transition from an M1-dominant inflammatory response to a pro-regenerative M2 phenotype after bacterial burden has been reduced. This phenotypic transition is primarily induced by incorporated therapeutic agents, including anti-inflammatory cytokines, small molecules, extracellular vesicles, and bioactive nanoparticles, rather than by the hydrogel matrix itself. The principal contribution of hydrogels is to provide localized retention and sustained release of these immunomodulatory payloads while minimizing systemic exposure (Wang et al., 2024).

The success of macrophage-targeting hydrogels is likely to depend as much on timing as on the therapeutic cargo itself. Suppressing inflammation too early may weaken host antimicrobial defence, whereas persistent M1 activation can hinder tissue regeneration. Rather than simply promoting M2 polarization, future systems should regulate the transition between inflammatory and reparative immune states in a manner that reflects the evolving biology of PJI and supports durable osseointegration.

### 6.2 Redox regulation

ROS play a dual role in PJI. Moderate ROS production contributes to bacterial killing during the early immune response, whereas persistent ROS accumulation caused by chronic inflammation and biofilm persistence induces oxidative stress, leading to mitochondrial dysfunction, osteoblast inhibition, endothelial injury, and excessive osteoclast activation (Marques-Carvalho et al., 2023). Therefore, therapeutic strategies should aim to restore redox homeostasis rather than indiscriminately eliminate ROS. Redox regulation is primarily mediated by incorporated antioxidants or ROS-scavenging agents, including cerium oxide nanoparticles, manganese dioxide, polydopamine, polyphenols, and other catalytic nanomaterials (Sun et al., 2023). Hydrogels enhance their therapeutic performance by providing localized retention, protecting bioactive molecules from premature degradation, and enabling sustained or ROS-responsive release within the inflammatory microenvironment. This localized delivery improves antioxidant efficiency while reducing systemic exposure and preserving physiological ROS levels required for normal immune function.

Although ROS-responsive hydrogels have shown promising results in preclinical studies, most evidence is derived from infected wound or osteomyelitis models rather than implant-associated PJI. Future studies should focus on optimizing redox-responsive release kinetics and validating their efficacy in clinically relevant PJI models, where balanced regulation of oxidative stress may facilitate both infection resolution and subsequent bone regeneration.

### 6.3 Osteogenesis-angiogenesis coupling

Successful reconstruction of the peri-implant interface requires coordinated osteogenesis and angiogenesis after infection has been controlled (Masters et al., 2022). Persistent inflammation and biofilm-associated oxidative stress impair osteoblast differentiation, inhibit endothelial cell function, and disrupt the coupling between bone formation and neovascularization, ultimately compromising osseointegration and implant stability (Yang and Peng, 2025). Therefore, regenerative strategies should promote both vascularization and bone repair rather than targeting either process alone. These regenerative effects are primarily mediated by incorporated bioactive agents, such as growth factors, bioactive ions, stem cells, extracellular vesicles, or osteoinductive nanoparticles. Hydrogels serve as localized delivery platforms that retain these payloads at the defect site, protect their biological activity, and enable sustained or sequential release (Zhang et al., 2022). Their ECM-like hydrated structure also provides a favorable microenvironment for cell infiltration and tissue remodeling, facilitating the coordinated progression of angiogenesis and osteogenesis during the healing process (Man et al., 2022).

The timing of regenerative therapy is as important as the therapeutic cargo itself. Osteogenic and angiogenic interventions are unlikely to succeed in the presence of persistent infection or unresolved inflammation, where bacterial activity and inflammatory mediators continue to disrupt tissue repair. Future hydrogel platforms should therefore align regenerative signalling with disease progression, introducing pro-regenerative cues only after effective infection control to maximize osseointegration and long-term implant stability.

### 6.4 Regulation of osteoclast activity

Excessive osteoclast activation is a major contributor to peri-implant bone loss in PJI. Persistent inflammation and biofilm-associated cytokines, particularly RANKL, TNF-α and IL-1β, stimulate osteoclast differentiation and bone resorption, disrupting the balance between bone formation and remodeling (Campbell et al., 2024). Therefore, limiting pathological osteoclast activity is essential for preserving peri-implant bone integrity and facilitating successful osseointegration. The inhibition of osteoclastogenesis is primarily mediated by incorporated therapeutic agents, including osteoprotegerin (OPG), bisphosphonates, anti-inflammatory molecules, and bioactive nanoparticles (Li et al., 2026). Hydrogels enhance their therapeutic efficacy by providing localized retention, sustained release, and prolonged exposure within the peri-implant microenvironment while reducing systemic adverse effects. In addition, controlled local delivery may help coordinate osteoclast inhibition with infection resolution and subsequent bone regeneration, thereby avoiding excessive suppression of physiological bone remodeling.

Current evidence is largely derived from bone defect and osteomyelitis models, leaving the effectiveness of osteoclast-targeting hydrogels in implant-associated PJI uncertain. Future studies should move beyond demonstrating reduced bone resorption and instead identify treatment windows that limit pathological osteolysis while preserving the physiological remodeling needed for osseointegration and long-term implant stability.

### 6.5 Emerging perspective: neuro-osteoimmune regulation

Neuro-osteoimmune regulation has recently emerged as a potential mechanism linking the nervous system, immune responses, and bone remodeling (Zhao et al., 2026). Neurotransmitters and neuropeptides may influence macrophage polarization, inflammatory signaling, angiogenesis, and osteogenesis, suggesting a possible role in peri-implant tissue repair. However, direct evidence supporting neuro-osteoimmune modulation in PJI remains limited. Most current studies involve conductive or electroactive hydrogels evaluated in bone regeneration or infectious bone defect models rather than implant-associated infections (Jing et al., 2023; Zheng et al., 2023). Therefore, although this concept offers an intriguing direction for future hydrogel design, its therapeutic relevance in PJI should currently be regarded as exploratory and requires further validation in clinically representative implant-associated infection models.

## 7 Comparison with conventional platforms

Hydrogels have emerged as promising local therapeutic platforms for PJI owing to their injectability, controlled release, and microenvironment-responsive properties. However, they are not the only option for local infection management. Conventional platforms, including polymethylmethacrylate (PMMA) bone cement, calcium sulfate carriers, implant coatings, nanoparticles, and electrospun scaffolds, each possess distinct advantages and limitations in terms of mechanical support, drug delivery, degradability, and clinical applicability. Therefore, the value of hydrogels should be assessed relative to these established approaches rather than in isolation. This section compares the material characteristics, therapeutic capabilities, and translational potential of major local delivery platforms, highlighting the clinical scenarios in which hydrogels offer distinct advantages and those in which alternative materials remain preferable. Such comparisons provide a more balanced framework for rational platform selection and future biomaterial design in PJI management.

The major local therapeutic platforms currently available for PJI differ substantially in their mechanical properties, drug-delivery characteristics, clinical maturity, and appropriate indications. Rather than replacing existing technologies, hydrogels should be considered within this broader therapeutic landscape. A scenario-based comparison of representative local therapeutic platforms is summarized in Table 2, providing an overview of their principal strengths, limitations, translational status, and preferred clinical applications.

**TABLE 2:** Scenario-specific comparison of local therapeutic platforms for PJI.

Platform	Major strengths	Major limitations	Mechanical role	Release characteristics	Most appropriate PJI scenarios	Clinical maturity
Hydrogels	Injectable; conformable; wet-interface adaptable; compatible with labile payloads; tunable degradation and responsive release	Low load-bearing capacity; washout or premature degradation; sterilization and storage sensitivity	Minimal unless combined with a rigid scaffold	Highly tunable; sustained, sequential, or stimulus-responsive	Prophylaxis coatings, DAIR adjuncts, irregular dead-space delivery, biologically active reconstruction	Promising; limited clinical products and indication-specific evidence
PMMA cement	High mechanical strength; established surgical familiarity; high local antibiotic loading	Nondegradable; heat of polymerization; removal often required; burst followed by low-level release	High temporary structural support	Initial burst with prolonged low release	Two-stage revision spacers and situations requiring temporary stability	Clinical standard in chronic PJI pathways
Calcium sulfate	Biodegradable; dead-space filling; high local antibiotic delivery; no planned removal	Rapid resorption or release; wound drainage; limited control of sensitive biologics	Low to moderate defect filling, not definitive fixation	Relatively rapid dissolution-mediated release	DAIR or revision dead-space management	Established adjunct with substantial clinical use
Nanoparticles/microspheres	High loading efficiency; intracellular or targeted delivery; responsive designs	Dispersion, clearance, aggregation, toxicity, and limited retention when used alone	None	Tunable at nano- or microscale	Best embedded within hydrogels or scaffolds for local retention	Predominantly preclinical
Electrospun scaffolds	Fibrous ECM-like architecture; higher structural integrity than soft hydrogels; surface functionalization	Poor injectability and conformity; fabrication and implantation complexity	Moderate scaffold support	Diffusion or degradation-controlled	Accessible bone defects and combined regenerative scaffolds	Preclinical to early translational
Permanent implant coatings	Direct anti-adhesion or antibacterial action at the implant surface; no separate local depot	Fixed composition; limited dose capacity; possible wear, cytotoxicity, or interference with osseointegration	Preserves implant structural role	Surface-limited and often non-reloadable	Prevention during primary or revision implantation	Some clinically used technologies; highly product specific

### 7.1 Hydrogels versus PMMA bone cement

PMMA bone cement remains the most widely used local therapeutic platform for PJI, particularly as an antibiotic-loaded spacer during two-stage revision arthroplasty (Hasandoost et al., 2020). Its major advantages include excellent mechanical strength, established surgical protocols, and the ability to provide temporary joint stability while delivering high local concentrations of antibiotics. These characteristics have made antibiotic-loaded PMMA the current clinical standard for managing chronic PJI (Jevnikar et al., 2024). Despite its widespread use, PMMA has several inherent limitations. As a nondegradable material, it generally requires surgical removal during staged revision procedures. In addition, polymerization generates heat that may limit the incorporation of heat-sensitive bioactive agents, and antibiotic release is often characterized by an initial burst followed by prolonged subtherapeutic concentrations. Residual PMMA may also serve as a potential surface for bacterial recolonization once antibiotic release declines.

Compared with PMMA, hydrogels offer greater flexibility as local drug-delivery platforms. Their injectable nature allows them to conform to irregular peri-implant spaces, while mild fabrication conditions permit encapsulation of diverse therapeutic payloads, including antimicrobial peptides, proteins, nanoparticles, and cells. Furthermore, tunable degradation and stimuli-responsive release enable more precise control of drug delivery than conventional PMMA systems. However, hydrogels cannot currently replace PMMA in many clinical settings because of their limited mechanical strength and relatively immature clinical evidence. Rather than competing directly, these two platforms should be regarded as complementary technologies. PMMA remains advantageous when temporary structural support is required, whereas hydrogels are particularly suitable for localized, multifunctional, and biologically responsive drug delivery, especially in applications emphasizing infection control and osteoimmune regulation rather than mechanical reconstruction.

### 7.2 Hydrogels versus calcium sulfate carriers

Calcium sulfate (CS) carriers are another established platform for local antimicrobial delivery in PJI. As biodegradable antibiotic carriers, they can fill peri-implant dead spaces while releasing high local concentrations of antibiotics, eliminating the need for secondary removal surgery. Their osteoconductive properties and extensive clinical experience have made them a commonly used adjunct in the management of implant-associated infections. Despite these advantages, calcium sulfate carriers exhibit relatively limited control over drug release. Antibiotics are typically released rapidly following implantation, resulting in an initial burst that may be followed by a rapid decline in local drug concentration. In addition, rapid material dissolution has been associated with prolonged wound drainage, local inflammation, and, in some cases, transient hypercalcemia (Abosala and Ali, 2020). Furthermore, calcium sulfate primarily functions as a passive drug carrier and offers limited flexibility for incorporating multiple therapeutic agents or responding to changes in the local pathological microenvironment.

Hydrogels provide several complementary advantages over calcium sulfate. Their highly hydrated polymer networks enable more precise regulation of degradation and release kinetics, facilitating sustained or stimuli-responsive delivery of antibiotics, antimicrobial peptides, immunomodulatory molecules, or regenerative factors. Injectable hydrogels also conform more readily to irregular peri-implant spaces and can accommodate a wider range of bioactive payloads under mild fabrication conditions. However, unlike calcium sulfate, most hydrogels lack intrinsic osteoconductivity and provide minimal mechanical support. Therefore, rather than replacing calcium sulfate, hydrogels may be particularly valuable when prolonged, programmable, or multifunctional local therapy is required, while calcium sulfate remains an effective option for straightforward biodegradable antibiotic delivery in appropriately selected clinical scenarios.

### 7.3 Hydrogels versus nanoparticles and microspheres

Nanoparticles and microspheres are widely investigated drug-delivery systems for the treatment of PJI because of their high loading capacity, tunable release profiles, and ability to encapsulate a broad range of therapeutic agents. Functional nanoparticles, including metallic nanoparticles, nanozymes, liposomes, and polymeric nanoparticles, can also provide additional antibacterial, antioxidative, or immunomodulatory activities. However, when administered alone, these particulate systems often exhibit limited local retention within the peri-implant environment. They may diffuse away from the target site, be rapidly cleared by body fluids, or undergo nonspecific uptake by surrounding tissues, thereby reducing local therapeutic efficacy (Indelli et al., 2021; Zhang Z. et al., 2025).

Hydrogels address these limitations by serving as a three-dimensional matrix that immobilizes nanoparticles or microspheres at the implant–tissue interface. The hydrated polymer network prolongs local retention, protects encapsulated particles from premature clearance, and enables sustained or stimuli-responsive release. In addition, injectable hydrogels conform to irregular peri-implant defects, ensuring more uniform distribution of nanoparticle-based therapeutics throughout the infected region. This combination preserves the unique biological functions of nanoparticles while improving their spatial localization and therapeutic duration.

Rather than representing competing technologies, hydrogels and nanoparticles are increasingly regarded as complementary platforms. Nanoparticles provide the primary therapeutic functions, such as antimicrobial activity, catalytic ROS regulation, or targeted drug delivery, whereas hydrogels optimize their local retention and release behavior. Consequently, hydrogel–nanoparticle composites have emerged as a promising strategy for integrating infection control with osteoimmune regulation. Future research should focus on improving manufacturing reproducibility, long-term safety, and validation in clinically relevant implant-associated PJI models to facilitate translation into clinical practice.

### 7.4 Hydrogels versus electrospun scaffolds

Electrospun scaffolds are widely used in bone tissue engineering because their fibrous architecture closely resembles the native ECM, providing favorable mechanical support and guidance for cell adhesion, migration, and osteogenic differentiation (Huang et al., 2024). Their high surface area also facilitates drug loading and sustained release, making them attractive for localized infection control and bone regeneration.

Compared with electrospun scaffolds, hydrogels offer superior injectability and conformability. Injectable hydrogels can readily fill irregular peri-implant spaces created during surgical debridement and establish intimate contact with infected tissues and implant surfaces, whereas electrospun scaffolds generally require preformed structures and are less adaptable to complex anatomical defects. Hydrogels are also more suitable for encapsulating cells, proteins, and other labile biomolecules under mild fabrication conditions.

However, electrospun scaffolds possess significantly greater mechanical strength and structural stability than hydrogels, making them more appropriate for large bone defects requiring mechanical support. Consequently, these two platforms should be viewed as complementary rather than competing technologies. Composite systems that integrate electrospun scaffolds with hydrogels may combine the mechanical advantages of fibrous scaffolds with the localized delivery and microenvironment-responsive capabilities of hydrogels, providing a promising strategy for simultaneous infection control and peri-implant bone regeneration.

### 7.5 Hydrogels versus conventional implant coatings

Conventional implant coatings, including silver-, antibiotic-, hydroxyapatite-, and DAC-based systems, are designed primarily to prevent early bacterial adhesion and biofilm formation on implant surfaces. Their principal advantage lies in acting directly at the implant–tissue interface during the initial postoperative period, thereby reducing the risk of bacterial colonization without substantially altering surgical procedures (Xiong et al., 2025). Several coating technologies have already progressed to clinical evaluation, demonstrating favorable translational potential.

Compared with conventional coatings, hydrogels offer greater flexibility in therapeutic design. Injectable or coating-type hydrogels can accommodate a broader range of bioactive payloads, including antimicrobial peptides, nanoparticles, cytokines, and growth factors, while enabling sustained or stimuli-responsive release. Their hydrated polymer networks also permit better adaptation to irregular peri-implant spaces and support multifunctional strategies that integrate infection control with osteoimmune regulation and tissue regeneration.

Nevertheless, conventional coatings and hydrogels are not mutually exclusive. Implant coatings are particularly suitable for prophylaxis against early bacterial adhesion, whereas hydrogels are more advantageous for managing established infection or delivering sequential therapeutic signals after debridement. Future strategies may combine durable implant coatings with hydrogel-based local delivery systems to provide continuous protection across different stages of PJI management, from infection prevention to tissue regeneration.

### 7.6 Clinical scenario-based platform selection

No single biomaterial platform is optimal for every stage of PJI management. Instead, platform selection should be guided by the clinical scenario, therapeutic objective, and local tissue condition. Conventional implant coatings are most suitable for preventing early bacterial adhesion during primary arthroplasty, whereas injectable hydrogels are particularly advantageous for DAIR procedures because they can conform to irregular peri-implant spaces and provide localized, sustained drug delivery (Pressato et al., 2023; Xiong et al., 2025). In contrast, antibiotic-loaded PMMA spacers remain the preferred option for two-stage revision owing to their mechanical stability and extensive clinical validation (Jevnikar et al., 2024). For patients with substantial bone loss, composite systems combining hydrogels with porous metallic scaffolds, calcium phosphate ceramics, or bone grafts may better integrate infection control with structural reconstruction (Chen et al., 2023).

These comparisons indicate that hydrogels should not be regarded as universal replacements for existing local therapeutic platforms. Their greatest strengths lie in localized retention, programmable release, and the ability to integrate antimicrobial, immunomodulatory, and regenerative therapies within a single platform. Future biomaterial design should therefore emphasize application-oriented strategies, selecting or combining platforms according to specific clinical needs rather than pursuing maximal multifunctionality. Such an approach is more likely to facilitate clinical translation and improve long-term outcomes for patients with PJI.

## 8 Evidence relevance and clinical integration

### 8.1 Evidence hierarchy and relevance to true PJI

The preclinical evidence base remains heterogeneous, and model relevance must be stated explicitly. Clinical PJI studies provide the most direct evidence, followed by implant-associated infection models that include a prosthesis or fixation device, a defined biofilm-forming inoculum, peri-implant tissue analysis, and assessment of implant stability or osseointegration. Osteomyelitis and infected bone-defect models can test antimicrobial and regenerative mechanisms, but they do not fully reproduce the prosthetic surface, retained implant, joint environment, or surgical decision pathways of PJI. Infected wound models are still more indirect and should be used only to illustrate material mechanisms such as responsive release, immune regulation, or biofilm disruption.

Representative systems are therefore classified in Table 1 as clinical PJI, implant-associated infection, infected bone defect or osteomyelitis, non-implant infection, or non-infected regeneration. This distinction prevents promising results from general wound or bone regeneration models from being presented as established PJI efficacy.

### 8.2 Surgical integration and intraoperative usability

Beyond biological performance, the clinical success of hydrogel-based therapies depends heavily on their compatibility with routine surgical practice. An ideal hydrogel should be easy to prepare, rapidly applied during surgery, and seamlessly integrated into established procedures such as DAIR, one-stage revision, or two-stage revision. Material properties including injectability, gelation time, adhesiveness, and conformability directly influence surgical handling and, ultimately, clinical adoption. Injectable hydrogels are particularly attractive because they can fill irregular peri-implant dead spaces created after debridement and establish intimate contact with infected tissues without requiring extensive modification of current surgical workflows (Carballo-Pedrares et al., 2026). Appropriate gelation kinetics are equally important: excessively slow gelation may result in material leakage or washout, whereas overly rapid gelation can hinder uniform application and precise placement. In addition, hydrogels should maintain stability in the presence of blood, irrigation fluids, and mechanical manipulation while remaining compatible with implant fixation and wound closure (Almawash et al., 2022).

From a translational perspective, ease of use may be as important as biological efficacy. Complex preparation procedures, multiple mixing steps, or highly operator-dependent application can limit widespread clinical implementation despite promising preclinical results (Correa et al., 2021).

### 8.3 Sterilization, storage, and manufacturing

The successful clinical translation of hydrogel-based therapies depends not only on biological efficacy but also on their manufacturability and product stability. Sterilization is a critical requirement for clinical use; however, commonly used methods such as gamma irradiation, ethylene oxide, and steam sterilization may alter polymer crosslinking, mechanical properties, degradation behavior, or the biological activity of encapsulated proteins, peptides, and cells (Galante et al., 2018). Therefore, sterilization strategies must be carefully selected according to both the hydrogel composition and the incorporated therapeutic payloads.

Storage stability represents another important challenge. Many multifunctional hydrogels contain biologically active molecules that are susceptible to degradation during long-term storage, often requiring refrigeration, lyophilization, or on-site preparation immediately before surgery (Carballo-Pedrares et al., 2026). Such requirements increase logistical complexity and may limit routine clinical use. Ready-to-use formulations with extended shelf life would therefore provide significant practical advantages.

In addition, large-scale manufacturing requires reproducible synthesis, batch-to-batch consistency, and compliance with Good Manufacturing Practice (GMP) standards (Correa et al., 2021). These considerations become increasingly challenging as hydrogel formulations incorporate multiple bioactive components or stimuli-responsive chemistries.

### 8.4 Regulatory considerations and clinical translation

Despite the rapid expansion of hydrogel research for PJI, only a limited number of systems have progressed to clinical evaluation. Clinical evidence remains concentrated on rapidly resorbable antibiotic-loaded implant coatings, particularly the DAC platform. A major barrier to translation is that most hydrogel platforms are combination products, integrating biomaterials with antibiotics, antimicrobial peptides, nanoparticles, growth factors, or cells. Such complexity increases regulatory requirements, as both the material and the incorporated therapeutic components must demonstrate safety, efficacy, and manufacturing consistency. In addition to standard biocompatibility testing, regulatory approval requires comprehensive evaluation of degradation products, release kinetics, sterility, batch-to-batch reproducibility, and long-term safety (Antich-Isern et al., 2021; Tian et al., 2022).

Another important limitation is the gap between preclinical studies and clinical practice. Many hydrogel systems have been validated in small-animal models of osteomyelitis or infected bone defects rather than clinically relevant implant-associated PJI models (Carli et al., 2016; Zhang et al., 2026). Moreover, standardized protocols for efficacy assessment, large-animal validation, and multicenter clinical trials remain scarce, making direct comparison among different hydrogel systems difficult.

## 9 Future directions

Over the past decade, hydrogel-based strategies for PJI have evolved from simple local drug delivery systems to multifunctional platforms capable of integrating infection control, osteoimmune regulation, and tissue regeneration (Zhang et al., 2026). As discussed in the preceding sections, many of the reported biological functions are primarily mediated by incorporated therapeutic agents, whereas hydrogels provide the material framework for localized retention, controlled release, and microenvironment-responsive delivery. Consequently, future advances are unlikely to arise from simply incorporating additional bioactive components but rather from optimizing hydrogel design according to specific clinical requirements.

Future hydrogel development should therefore emphasize rational design, stage-specific therapy, and translational feasibility. This includes matching material properties to different clinical scenarios, coordinating antimicrobial and regenerative interventions over time, and improving compatibility with surgical workflows, manufacturing, and regulatory requirements. In this section, we discuss the key directions that may guide the next-generation of hydrogel systems toward more effective and clinically applicable therapies for PJI.

### 9.1 From maximal multifunctionality to rational design

The development of hydrogel systems for PJI has increasingly focused on incorporating multiple therapeutic functions into a single platform. Antibacterial agents, AMPs, antioxidants, immunomodulators, angiogenic factors, and osteogenic cues are often combined to address the multifactorial nature of PJI. While such multifunctional systems have demonstrated promising preclinical efficacy, increasing material complexity does not necessarily translate into greater clinical benefit. Additional functional components may complicate material synthesis, reduce manufacturing reproducibility, increase regulatory challenges, and make it more difficult to control release kinetics and therapeutic interactions (Carballo-Pedrares et al., 2026). Future hydrogel development should therefore shift from pursuing maximal multifunctionality toward rational, application-driven design. Rather than integrating every available therapeutic modality, hydrogel systems should be tailored to specific clinical scenarios and unmet clinical needs. For example, a prophylactic implant coating may primarily require short-term antibacterial activity, whereas a hydrogel for DAIR procedures should emphasize local retention and sustained antimicrobial delivery (Romanò et al., 2015; De Meo et al., 2023). In contrast, hydrogels intended for chronic PJI with extensive bone loss should prioritize sequential osteoimmune regulation and tissue regeneration following effective infection control (Zhang R. et al., 2025). This strategy also requires balancing biological performance with translational feasibility. Simplified formulations with well defined compositions, reproducible manufacturing processes, and predictable release profiles are more likely to progress toward clinical application than highly complex systems with uncertain regulatory pathways. Consequently, the next-generation of hydrogel platforms should be designed according to the principle of “necessary functionality rather than maximal functionality,” ensuring that each incorporated component provides a clear therapeutic advantage while maintaining clinical practicality, scalability, and safety.

### 9.2 Stage-adaptive and spatiotemporal therapy

PJI is a progressive disease in which the pathological microenvironment changes from acute infection to persistent inflammation and subsequent tissue repair (Patel, 2023). Therapeutic strategies that rely on a single intervention or concurrent delivery of multiple bioactive agents may therefore fail to match the changing biological requirements at different stages of disease progression. Instead, future hydrogel systems should be designed to provide stage-adaptive and spatiotemporally controlled therapy, matching therapeutic interventions to the evolving biological requirements of the peri-implant microenvironment. In the early stage of infection, hydrogel platforms should prioritize rapid bacterial eradication and biofilm disruption through localized delivery of antimicrobial agents. As the infectious burden decreases, subsequent release of anti-inflammatory molecules or immunomodulatory agents may facilitate the transition from persistent M1-dominated inflammation toward a pro-regenerative osteoimmune environment. During the later repair phase, sequential delivery of angiogenic and osteogenic factors can promote vascular reconstruction, bone regeneration, and restoration of implant osseointegration. Such temporal coordination more closely mimics the natural healing process than the concurrent administration of all therapeutic components. Achieving this strategy requires hydrogels capable of programmable degradation, controlled release, or responsiveness to pathological cues such as ROS, pH changes, or bacterial enzymes. Rather than serving as passive drug carriers, future hydrogels should function as dynamic therapeutic platforms that coordinate infection control with osteoimmune regulation and tissue regeneration. This stage-specific approach may improve therapeutic efficiency while minimizing unnecessary drug exposure and reducing the complexity associated with indiscriminate multifunctional delivery.

### 9.3 Precision biomaterials and personalized therapy

PJI is a heterogeneous disease in which pathogen characteristics, host immune responses, implant conditions, and bone defects vary considerably among patients (Patel, 2023). These variations make it unlikely that a single hydrogel platform can meet the therapeutic requirements of all clinical settings. Future hydrogel development should focus on precision biomaterials that are adapted to the pathological and clinical features of individual patients. Material composition, therapeutic payloads, and release kinetics could be selected according to pathogen identification, antimicrobial susceptibility, local inflammatory activity, and the extent of peri-implant bone loss. Advances in three-dimensional (3D) printing, computational modeling, and artificial intelligence (AI)-assisted materials design may support this process by improving control over hydrogel composition, degradation, and drug release (Li et al., 2024). These tools may help align material performance with the changing therapeutic priorities of different stages of PJI management. However, personalized hydrogel systems also introduce challenges related to manufacturing complexity, quality control, regulatory approval, and cost-effectiveness. Future research should therefore balance individualized therapeutic strategies with standardized production processes to ensure both clinical efficacy and practical feasibility. By integrating precision medicine with rational biomaterial design, next-generation hydrogel platforms may provide more effective, patient-centered solutions for the management of PJI.

### 9.4 Standardized evaluation for clinical translation

A major barrier to the clinical translation of hydrogel-based therapies is the lack of standardized preclinical evaluation. Most published studies use different hydrogel formulations, animal models, bacterial strains, outcome measures, and follow-up periods, making direct comparison among studies difficult (Andresen et al., 2026; Zhang et al., 2026). Furthermore, many hydrogel systems are validated in infected wound, osteomyelitis, or bone-defect models rather than clinically relevant implant-associated PJI models, limiting the reliability of conclusions regarding their translational potential. Future studies should adopt more standardized and clinically representative evaluation strategies. Preclinical assessment should include mature biofilm models, implant-associated infection models, and large-animal studies that better replicate the surgical environment, mechanical loading, and host responses encountered in clinical PJI. In addition to antibacterial efficacy, evaluation should incorporate osteoimmune regulation, osseointegration, implant stability, degradation behavior, local and systemic biosafety, and long-term functional outcomes. Standardized reporting of material properties, release kinetics, and experimental protocols would further improve reproducibility and facilitate meaningful comparisons across studies. Equally important, future research should strengthen the connection between laboratory investigations and clinical evidence. Multicenter collaborations, consensus evaluation criteria, and well-designed prospective clinical trials will be essential for establishing the safety, efficacy, and practical value of hydrogel-based therapies. Such standardized translational frameworks will help accelerate the progression of promising hydrogel systems from experimental biomaterials to evidence-based therapeutic options for PJI.

## 10 Conclusion

PJI combines implant-associated biofilm persistence with chronic immune activation, oxidative stress, bone loss, and failed osseointegration. Durable treatment therefore requires coordinated control of infection and reconstruction of the peri-implant environment rather than escalation of antibacterial intensity alone.

A central premise of this review is that biological effects should not be attributed indiscriminately to the hydrogel platform. Matrix-intrinsic functions, payload-derived activities, and hydrogel-enabled contributions must be distinguished. Hydrogels provide their clearest advantage when they improve local retention, conform to irregular peri-implant spaces, protect sensitive therapeutics, adapt to wet implant surfaces, or coordinate sustained, sequential, or pathology-responsive release.

Hydrogels are not universal replacements for PMMA, calcium sulfate, structural scaffolds, nanoparticles, or permanent coatings. Their value depends on the clinical task, and their limited mechanical strength, variable model relevance, sterilization sensitivity, manufacturing complexity, and regulatory burden remain substantial barriers. Future progress should prioritize indication-driven formulations, clinically representative PJI models, standardized evaluation, and surgical usability. Systems that deliver necessary rather than maximal functionality are most likely to become practical adjuncts to established PJI treatment.
